# Monitoring of Insecticide Resistance Mutations and Pathogen Circulation in Sand Flies from Emilia-Romagna, a Leishmaniasis Endemic Region of Northern Italy

**DOI:** 10.3390/v15010148

**Published:** 2023-01-03

**Authors:** Sofia Balaska, Mattia Calzolari, Annalisa Grisendi, Mara Scremin, Michele Dottori, Konstantinos Mavridis, Romeo Bellini, John Vontas

**Affiliations:** 1 Institute of Molecular Biology & Biotechnology, Foundation for Research & Technology Hellas, 70013 Heraklion, Greece; 2 Department of Biology, University of Crete, 70013 Heraklion, Greece; 3 Istituto Zooprofilattico Sperimentale della Lombardia e dell’Emilia Romagna (IZSLER) “B. Ubertini”, 25124 Brescia, Italy; 4 Centro Agricoltura Ambiente (CAA) “Giorgio Nicoli”, Via Sant’Agata 835, Crevalcore, 40014 Bologna, Italy; 5 Pesticide Science Lab, Department of Crop Science, Agricultural University of Athens, 11855 Athens, Greece

**Keywords:** *Phlebotomus*, *Leishmania*, phleboviruses, pyrethroid resistance, *kdr* mutations, molecular diagnostics

## Abstract

The continuously expanding distribution of sand flies, proven vectors of *Leishmania* and of several phleboviruses, is a growing public health issue in Europe. Especially in Italy, visceral leishmaniasis (VL) is occurring with increasing incidence northward, in previously non-endemic provinces. Around the globe, disease elimination efforts largely focus on sand fly vector insecticidal control, often leading to the development of resistance. In Emilia-Romagna (ER), northern Italy, insecticides are heavily applied for agricultural and mosquito control, but not specifically against sand flies. Here, we investigated the sand fly species composition in certain environmental settings in ER provinces and monitored the presence of pyrethroid resistance mutations and pathogen circulation. *Phlebotomus perfiliewi*, a dominant vector of *Leishmania infantum*, was detected almost exclusively in the region. No mutations in the voltage-gated sodium channel gene, e.g., knock-down resistance mutations I1011M, L1014F/S, V1016G, or F1020S, were recorded. Pathogen monitoring revealed that almost 40% of the tested sand fly pools were positive for *Leishmania*, while the presence of Toscana and Fermo phleboviruses was also observed in much lower frequencies (≤3% positive pools). Regular epidemiological and entomological monitoring, alongside resistance surveillance, is highly recommended to ensure the sustainability and efficiency of vector control interventions.

## 1. Introduction

Leishmaniasis is second only to malaria among the deadliest protozoan diseases globally. The prevalence of leishmaniasis in Europe, largely under-reported, counts for less than 2% of the global prevalence [1]. However, in the last decades, the re-emergence of vector-borne diseases has been witnessed across the continent, making leishmaniasis a growing public health concern, especially for Mediterranean countries [2,3].

In Italy, as of 1990 in particular, canine leishmaniasis, caused by *Leishmania infantum* (Kinetoplastida: Trypanosomatidae), has been expanding northward into previously non-endemic regions, with an approximately 10-fold increase in seroprevalence rates (2.1% to 21.6%) since 2009 [4,5]. *Phlebotomus perniciosus* and *P. perfiliewi* are considered the dominant sand fly (Diptera: Psychodidae) vector species in the country [6]. Sand flies’ broadening distribution patterns, mainly attributed to global warming, environmental modifications, and their remarkable ecological plasticity, facilitate pathogen circulation and the establishment of new endemic disease foci [4].

Interestingly, the impact of sand fly distribution on public health is not restricted to leishmaniasis transmission. Phlebotomines are additionally incriminated as vectors of a variety of phleboviruses (family: Phenuiviridae, genus: Phlebovirus), such as Toscana virus (TOSV; a causative agent of neuroinvasive infections in humans), sand fly Naples and Sicilian virus, Fermo virus (FERMV), etc. [7,8,9,10]. Even though the circulation of the aforementioned viruses in the Mediterranean basin is well-evident, their public health significance remains greatly disregarded, as scarce information exists on their epidemiology and transmission cycles [11].

In the absence of preventive human vaccines and safe therapeutic drugs against leishmaniasis, apart from case management (early detection and treatment) [12], sand fly control, either by utilizing synthetic insecticides or by managing environmental habitats, stands as the cornerstone of disease elimination efforts in many endemic countries [13]. Emilia-Romagna (ER; north-eastern Italy), a leishmaniasis endemic region, is extensively cultivated, with the annual usage of pesticides for agricultural purposes reaching approximately 1400 tons [14]. Besides this, given the touristic exploitation of the region, during the last decades, pyrethroid sprayings (principally deltamethrin and permethrin, according to the European Union directive 528/2012 [15]) have been implemented against mosquito species, such as *Culex pipiens* and *Aedes albopictus*, as part of regional vector control programs and/or household-level interventions [16,17]. Although, so far, no targeted sand fly insecticidal control program has been applied in the country, it is highly anticipated that sand fly populations in ER provinces have been focally exposed to insecticides in areas where leishmaniasis is co-endemic with mosquito-borne diseases and in those proximal to agricultural areas.

Insecticide resistance, often fostered by the prolonged and excessive use of insecticides, critically impedes chemical control interventions against pests of agricultural and medical importance [18]. Regarding sand flies, information in the literature on the response profile of wild populations to insecticides around the globe remains seriously limited. However, recent phenotypic data (bioassays) and molecular analyses (e.g., detection of *knock-down resistance* (*kdr*) mutations L1014F/S in the voltage-gated sodium channel (*VGSC*) gene related to loss of sensitivity to pyrethroids) of dominant sand fly vector species, wild populations originating from countries with the highest leishmaniasis burden in north-eastern Asia and the Middle East, revealed that insecticide resistance in sand flies poses an up-coming and alarming issue [19].

Hence, regular monitoring of local sand fly populations constitutes a required precondition of integrated control campaigns, especially when incipient resistance, which could be missed by bioassays, needs to be detected as early as possible. The objective of the present study was to compile surveillance data from sand fly field collections in three ER provinces regarding (1) the sand fly species composition; (2) the presence of known pyrethroid resistance molecular markers (e.g., target-site mutations I1011M, L1014F/S, V1016G and F1020S in the *VGSC*); and (3) the *Leishmania* load, as well as the possible sand fly-borne phleboviruses, in an epidemiologically relevant macroarea.

## 2. Materials and Methods

### 2.1. Sampling Areas, Sand Fly Collection, and Sample Handling

**Figure 1:**
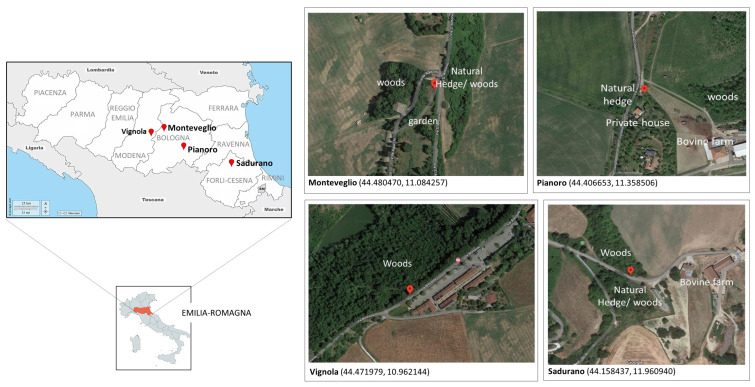
Sand fly sampling locations in Emilia-Romagna provinces, northern Italy. Red pins correspond to the study sites, whose coordinates are given in parentheses (X, Y). The base layers of the left panel’s maps were obtained from d-maps.com [21], and sampling locations’ screenshots were obtained from Google maps (accessed on 22 December 2022).

Multiple sand fly samplings were performed at four georeferenced sites in Vignola (Modena province; MO—1 sampling), Monteveglio and Pianoro (Bologna province; BO—8 samplings), and Sadurano (Forlì Cesena province; FC—1 sampling) in the Emilia-Romagna (ER) region between July and September 2021. Locations were selected in the interface between semi-natural environments (woods or hedges), agricultural areas/cultivated fields, and urbanized territories (streets, villas) (Figure 1; Table 1).

Sandflies were collected overnight using CDC miniature light traps baited with dry ice, set before 5 p.m. and removed at 7 a.m. the day after. The collection bags were refrigerated and transferred to the laboratory, where, prior to any further handling/analysis, they were anesthetized at 4 °C. Males were separated from females, and engorged females were removed. Specimens that were not immediately processed were conserved at −80 °C. A sub-sample of the collected sand flies was identified morphologically, using a light microscope and specific morphological keys [20]. Females were either (i) group in pools comprised of approximately 50–100 specimens from the same sampling location and collection date and submitted to molecular analysis for pathogen detection (manipulation of the females was kept to a minimum to optimize pathogen detection); or (ii) individually stored (60 sand fly individuals from each sampling location) for molecular verification of species and genotyping of *kdr* mutations.

### 2.2. Genetic Material Extraction from Sand Flies

Genomic DNA was extracted from 240 individual female sand flies (60 specimens from each sampling location) using the DNazol reagent according to the manufacturer’s instructions (Invitrogen, Carlsbad, CA, USA). For pathogen detection, pools of sand flies were homogenized using a pellet pestle, and DNA and RNA were automatically extracted using BioSprint 96 (Qiagen, Germany); RNA was retro-transcribed (Super Script II, Invitrogen, Carlsbad, CA, USA). The quantity and purity of DNA/RNA were assessed using a NanoDrop 2000c spectrophotometer (Thermo Scientific, Waltham, MA, USA).

### 2.3. Molecular Identification of Species and Genotyping of Mutations in the Voltage-Gated Sodium Channel (VGSC)

Discrimination of the sand fly species relied on the PCR amplification of a 700 bp mitochondrial cytochrome oxidase subunit I (*COI*) genomic fragment, using primers LCO1490 and HCO2198 [22,23] and *Taq* DNA polymerase (EnzyQuest, Heraklion, Greece). The applied thermal protocol was as follows: 94 °C for 2 min, 35 cycles × (94 °C for 45 s, 50 °C for 30 s, 72 °C for 45 s), 72 °C for 10 min. After agarose gel visualization of a small PCR product quantity (5 μL), the rest was purified using the Nucleospin PCR & Gel Clean-Up Kit (Macherey Nagel, Dueren, Germany) and then subjected to Sanger sequencing (GENEWIZ, Azenta Life Sciences, Griesheim, Germany) using the LCO1490 primer and BLASTn analysis.

The presence of *kdr* mutations associated with resistance to pyrethroid insecticides and previously detected in sand flies (L1014F/S) and/or other insect species (I1011M, V1016G, and F1020S) populations was monitored by genotyping the *VGSC* domain IIS6. The genomic sequence was amplified by *Taq* DNA polymerase (EnzyQuest, Heraklion, Greece) using primers Vssc8F and Vssc1bR, as described in Gomes et al., 2017 [24]. The DNA template (2 μL) used in this diagnostic assay consisted of a mixture of genomic DNAs extracted individually from up to 5 sand flies of the same species and sampling location. The pooled DNA included 1.5 μL of each individual gDNA and ddH_2_O up to 10 μL. The reaction’s thermal conditions were as follows: 94 °C for 2 min, 35 cycles × (94 °C for 45 s, 56 °C for 30 s, 72 °C for 30 s), 72 °C for 10 min. The approximately 400 bp generated PCR fragments, after visualization in agarose gel, were purified using the Nucleospin PCR & Gel Clean-Up Kit (Macherey Nagel, Dueren, Germany) and then subjected to Sanger sequencing (GENEWIZ, Azenta Life Sciences, Germany) using the Vssc8F primer. Sequences were analyzed using the sequence alignment editor BioEdit 7.2 (https://bioedit.software.informer.com/7.2/). Reference *VGSC* partial genomic sequences were obtained from GenBank for *Phlebotomus papatasi* (MH401419.1) and *P. perfiliewi* (MG779187.1)

### 2.4. Pathogen Detection

Detection of the *Leishmania* parasite in female sand fly pools relied on a TaqMan quantitative PCR (qPCR) assay targeting a 122 bp fragment of the kinetoplast DNA, as described by Galletti et al., 2011 [25].

A one-step Reverse Transcriptase PCR (RT-PCR) assay was performed to investigate the possible presence of phleboviruses in pools of sand fly samples, targeting a 370 bp nucleotide region of the S genomic segment, according to Lambert et al., 2009 [26]. The amplicons obtained with the pan-phlebo-PCR were sequenced and subjected to BLAST analysis for virus identification, based on GenBank. TOSV presence was monitored via specific real-time PCR, as described by Perez-Ruiz et al., 2007 [27].

## 3. Results

### 3.1. Sand Fly Collections and Species Identification

A total of *N* = 35,706 sand flies were collected from the four sampling locations, with 86.6% of them originating from Monteveglio and Pianoro (BO, eight samplings). A sub-group of *n*_1_ = 1644 samples was identified molecularly by species, relying on the *COI* marker. *Phlebotomus perfiliewi* was recorded almost exclusively (overall percentage: 99.2%) in all sampling locations, apart from four *P. perniciosus* individuals from Monteveglio (BO). In *n*_2_ = 240 *Phlebotomus* samples (out of the aforementioned *n*_1_), 60 from each location grouped into pools (p) of 5 individuals, the *VGSC* IIS6 region was genotyped for the presence of any pyrethroid resistance mutations. The investigation of *Leishmania* and phlebovirus load in the collected populations was conducted in *n*_3_ = 11,965 female sand flies (out of the *N Phlebotomus* samples) grouped into P = 132 pools of 50–100 individuals from the same sampling location and collection date (Table 1).

### 3.2. Monitoring of Knock-Down Resistance (kdr) Mutations

Sequencing of the *VGSC* domain IIS6 to monitor the occurrence of target-site mutations conferring resistance to pyrethroid insecticides revealed the presence of the wild-type allele TTA (leucine) at position 1014 in all the analyzed samples, *n*_2_ = 240 (Table 1). Similarly, wild-type codons were detected in all the other three *VGSC* loci investigated, i.e., ATT (isoleucine) at 1011, GTG (valine) at 1016, and TTC (phenylalanine) at 1020.

### 3.3. Detection of Leishmania and Phleboviruses

A total of 132 pools (P) of *Phlebotomus* sp. sand flies (*n*_3_ = 11,965 specimens, in groups of 50–100) were screened for the presence of *Leishmania* parasites and for TOSV and FERMV. Monitoring resulted in a total of 51 pools (38.6%) positive for *Leishmania*, out of which 45 were gathered from Monteveglio and Pianoro (BO). A lower percentage of pools was found to harbor TOSV or FERMV infection: 2.3% and 3%, respectively. Notably, co-infection with *Leishmania* and TOSV was recorded in a pool from Monteveglio (Table 1).

## 4. Discussion

The ongoing northward spread of visceral leishmaniasis (VL) in Italy highlights the necessity of seasonal surveillance programs, encompassing epidemiological and entomological parameters to alleviate disease transmission risk spatiotemporally. In support of this requirement, here we surveyed accordingly (semi-)rural areas close to small animal farms and/or cultivated fields and human residences in the Bologna, Modena, and Forli-Cesena provinces in Emilia-Romagna (ER), an important VL focus in the country.

*Phlebotomus perfiliewi*, consistently ranking as the most widespread sand fly species in ER, especially in the hilly central parts, was the dominant species documented in our collected samples. *P. perniciosus*, the main leishmaniasis vector in Italy, followed with a low percentage (<1%), in line with past literature data [28]. This finding could be possibly attributed to microhabitat preferences and/or the slightly different seasonal dynamics the two species exhibit. Indications of these species, along with *P. neglectus*, colonizing northern-latitude European regions beyond the confined endemic ones [5,29] raise concerns regarding the aggravation of the leishmaniasis epidemiological scenario on the continent.

The presence of the *Leishmania* parasite was recorded in approximately 40% of the tested sand fly pools. Previous surveillance studies, during 2018–2020, showed infection rates of <17% (examining pools of 50 females, in contrast to pools of 50–100 here) [10]. *L. infantum* has been exclusively documented in the surveyed regions in the past, based on specific molecular typing, implying that the isolates herein also belong to this species. In 2012, after almost 30 years, human leishmaniasis re-emerged in northern Italy, with *P. perfiliewi* “driving” several outbreaks (of canine leishmaniasis, as well) so far [29,30,31,32]. Interestingly, Calzolari et al., 2019 [31] described that *L. infantum* strains circulating in ER differ genetically based on their reservoir host (humans or dogs), suggesting that two distinct but overlapping *Leishmania* transmission cycles may occur in the region. The high infection prevalence marked here conjugated with *P. perfiliewi* abundance depicts the active risk of leishmaniasis transmission, potentially among both humans and dogs. Besides this, the co-circulation of sand-fly-borne phleboviruses, i.e., the Toscana (TOSV) and Fermo (FERMV) viruses, in the region is again attested to here. Apart from the importance of such viruses in human health per se, their potential to enhance *Leishmania* infection when both are present inside the host has been proposed [33], yet possible interactions of the two pathogens have been insufficiently investigated.

Regarding insecticide resistance, the application of molecular diagnostic tools revealed the absence of known knock-down resistance (*kdr*) mutations in the analyzed populations from ER. To the best of our knowledge, this is the first attempt to monitor resistance by the application of molecular diagnostics in sand flies originating from Italy. In the past, phenotypic profiling of insecticide resistance in important vector species populations (i.e., *P. perfiliewi*, *P. perniciosus*, and *P. papatasi*) from central and southern Italy indicated susceptibility to pyrethroid and/or acetyl-cholinesterase inhibitor insecticides [34,35]. Globally, investigations into the insecticide resistance status of sand fly populations and the background molecular mechanisms remain narrow. Nevertheless, resistance against widely used insecticides, such as DDT and pyrethroids, has arisen in countries with high disease endemicity and a history of immense insecticidal pressure for medical or agricultural purposes, such as India, Turkey, Iran, etc. Interestingly, mutations at the voltage-gated sodium channel (*VGSC*), L1014F and L1014S, conferring resistance to pyrethroids have been recorded focally in populations from India, Sri Lanka, and Turkey [19].

Worldwide sand fly chemical control is usually integrated into mosquito control programs, rather than being directly targeted [13]. Presumably, in the environmental settings we selected for samplings (semi-natural environments with woods, hedges, farms, cultivated fields, villas, and streets), the selection pressure from insecticide applications has been rare or absent. No regional spraying programs have operated there, and the use of insecticides at the household/farm level might be limited, possibly accounting for the absence of *kdr* mutations.

Field studies have revealed the restricted dispersal patterns (spatial movements) of some sand fly species in their distribution areas [36,37], indicating that any resistance trait could be focally present and, thus, difficult to detect in occasional field samplings in wider peridomicile settings. The possibility that pyrethroid resistance, either target-site or detoxification-based, may occur in the examined populations cannot be excluded. It is noteworthy that, especially in ER, but also in neighboring regions of northern Italy, pyrethroid resistance has been frequently recorded, phenotypically and molecularly, in *Ae. albopictus* and *Cx. pipiens* mosquito populations, probably due to urban chemical interventions conducted locally at the regional (by Municipalities) and/or household level [38,39].

The development of additional molecular diagnostic markers, to more reliably capture incipient insecticide resistance in sand flies, requires further molecular studies and, therefore, the availability of genomic resources (e.g., annotation of P450 monooxygenases and other detoxification genes, phylogenomic analyses, etc.). Genome sequencing of important sand fly vector species would greatly open up the possibility for efficient insecticide resistance management tools [39].

## 5. Conclusions

The Emilia-Romagna region, an epidemiologically important visceral leishmaniasis epicenter of Italy, receives heavy synthetic insecticide loads on an annual basis as part of both agricultural and public health interventions. Thus, monitoring for sandfly species, pathogen circulation, and insecticide resistance traits in this area is of major importance. In our study, co-circulation of *Leishmania* and sand-fly-borne phleboviruses was demonstrated in local populations, in line with previous findings of regional surveillance studies. Molecular analysis of pyrethroid resistance traits in *P. perfiliewi* populations, the dominant sand fly species of the region and a major *L. infantum* vector, revealed no occurrence of knock-down resistance mutations. However, given the selection of insecticide resistance in several sand fly populations from leishmaniasis endemic countries, systematic monitoring via the deployment of molecular markers and/or phenotypical assessment is highly recommended to facilitate early diagnosis of incipient resistance and assist with the application of evidence-based local control interventions.

## Figures and Tables

**Table 1:** Sand fly sampling locations in ER, species composition, and monitoring of *kdr* mutations and pathogen presence.

Location— Province (X, Y)	Date	Environmental Settings	*N*	Species Composition (%)				*kdr* L1014F/S			Pathogen Detection			
				*n*_1_	*perfi*	*perni*		*n*_2_ (p)	L (%)		P (*n*_3_)	*Leish*	TOSV	FERMV
Monteveglio—BO (44.480470, 11.084257)	July 2021	Abandoned villa in farmland, with sparse trees/shrubs, vineyards	18,579	512	99.2	0.8		60 (12)	100		59 (5462)	24	0	1
Pianoro—BO (44.406653, 11.358506)	July 2021	Inhabited villa in cattle pasture, sparse trees, grassland	12,349	852	100	0		60 (12)	100		49 (4562)	21	1	2
Sadurano—FC (44.158437, 11.960940)	August 2021	Hilly area proximal to farm	1323	122	100	0		60 (12)	100		9 (441)	2	2	1
Vignola—MO (44.471979, 10.962144)	September 2021	Area between villas, vineyards	3455	158	100	0		60 (12)	100		15 (1500)	4	0	0

ER, Emilia-Romagna; BO, Bologna; FC, Forli-Cesena; MO, Modena; (X,Y), coordinates of sampling location; *N*, total number of collected sand flies per location; *n*_1_, total number of specimens identified molecularly by species; *perfi*, *Phlebotomus perfiliewi*; *perni*, *Phlebotomus perniciosus*; *n*_2_, total number of specimens genotyped for *kdr* mutations in pools; p, number of pools into which *n*_2_ specimens were divided; *kdr*, knock-down resistance; L, susceptible wild-type allele (leucine); F, resistant mutant allele (phenylalanine); S, resistant mutant allele (serine); P, number of pools into which *n_3_* specimens were divided; *n*_3_, total number of specimens analyzed for the presence of pathogens in pools; *Leish*, *Leishmania*; TOSV, Toscana virus; FERMV, Fermo virus.

## Data Availability

Not applicable.
